# Multifactorial Pulmonary Hypertension: Converging Roles of Sarcoidosis, Heart Failure With Reduced Ejection Fraction, and Chronic Thromboembolic Disease

**DOI:** 10.7759/cureus.108187

**Published:** 2026-05-03

**Authors:** John Bajouka, Christopher Matti, Ghaid Touza, Keyur Patel, Ziad R Affas

**Affiliations:** 1 Internal Medicine, Henry Ford Health System, Southfield, USA; 2 Medicine, Wayne State University School of Medicine, Detroit, USA; 3 Cardiology, Henry Ford Health System, Southfield, USA

**Keywords:** chronic thromboembolic pulmonary hypertension (cteph), heart failure with reduced ejection fraction, multifactorial pulmonary hypertension, multiorgan sarcoidosis, right heart catheterization

## Abstract

Pulmonary hypertension (PH) is a multifactorial condition associated with elevated pulmonary arterial pressures. Treatment focuses on the identification and treatment of underlying causes. Here, we present the case of a 50-year-old man with advanced pulmonary sarcoidosis, recent pulmonary embolisms, and heart failure with reduced ejection fraction (HFrEF) of 35% who was found to have severe PH. This case describes a 50-year-old man with severe, multifactorial PH arising from three distinct World Health Organization (WHO) classifications. The patient, who has a history of advanced pulmonary sarcoidosis and HFrEF, presented with gastrointestinal bleeding and hemodynamic instability. Diagnostic workup, including a right heart catheterization, revealed significantly elevated pressures, including a mean pulmonary arterial pressure of 71 mmHg and a transpulmonary gradient of 54 mmHg. The complexity of his condition stems from the overlap of Group 2 PH (venous congestion from HFrEF), Group 4 PH (chronic thromboembolic disease), and Group 5 PH (sarcoidosis-related fibrosis and vascular compression).

Management of such mixed-etiology PH requires a highly tailored approach. For the Group 2 component, treatment focuses on goal-directed medical therapy (GDMT) for heart failure, including beta-blockers and sodium-glucose cotransporter 2 (SGLT2) inhibitors. The Group 4 component necessitates long-term anticoagulation and evaluation for surgical endarterectomy or riociguat therapy. Finally, the Group 5 component, driven by sarcoidosis, requires controlling systemic inflammation. As this case illustrates, when PH is multifactorial and refractory to standard interventions, lung transplantation remains the definitive consideration for long-term survival.

## Introduction

Pulmonary hypertension (PH) is a progressive hemodynamic syndrome characterized by elevated pulmonary arterial pressures that lead to right ventricular failure and increased mortality [1]. The World Health Organization (WHO) classifies PH into five groups based on underlying pathophysiology. These include pulmonary arterial hypertension (Group 1), left heart disease (Group 2), chronic lung disease (Group 3), chronic thromboembolic disease (Group 4), and unclear mechanisms (Group 5) [2]. While this framework provides a foundation for diagnosis and management, clinical practice often reveals multiple pathologies that converge to create a complex hemodynamic profile [3].

The global burden of PH is largely driven by Group 2 disease. This group affects up to 40-75% of patients with heart failure with reduced ejection fraction (HFrEF) [4]. In contrast, Group 5 PH, particularly sarcoidosis-associated pulmonary hypertension (SAPH), presents a unique challenge due to its multifactorial nature. The prevalence of PH in sarcoidosis varies widely, from 2% to 74%, depending on disease severity, diagnostic method, and the extent of pulmonary involvement. This variability reflects the diverse mechanisms underlying SAPH, including parenchymal fibrosis, hypoxic vasoconstriction, granulomatous vasculopathy, and pulmonary venous involvement [5].

When further compounded by chronic thromboembolic pulmonary hypertension (CTEPH), which develops in approximately 0.5-4% of patients following acute pulmonary embolism, the multifactorial pulmonary vascular insults emerge [2]. Although apixaban was initiated two weeks prior to admission, the persistence of organized vascular lesions and the severity of right ventricular remodeling indicated a chronic thromboembolic pathology (Group 4) rather than a purely acute presentation. The coexistence of postcapillary congestion (Group 2), mechanical obstruction (Group 4), and inflammatory and fibrotic remodeling (Group 5) creates a severe mixed pre- and postcapillary phenotype with significant diagnostic and therapeutic implications [2,4,5].

Diagnosing and managing multifactorial PH remains a major clinical challenge. Standard treatment algorithms are typically designed around a single dominant WHO group; however, in multifactorial disease, effective management requires a mechanism-directed approach that addresses each contributing pathology [6]. We present a case of severe multifactorial PH in a patient with advanced pulmonary sarcoidosis, CTEPH, and HFrEF, highlighting the importance of multidisciplinary management.

This work was previously presented as a meeting abstract at the American Thoracic Society International Conference on 05/17/2025 and published in the American Journal of Respiratory and Critical Care Medicine.

## Case presentation

A 50-year-old man with a history of advanced stage 4 pulmonary sarcoidosis on home two liters of oxygen and HFrEF presented with two days of melena. Two weeks prior, he had been initiated on apixaban for acute non-massive pulmonary embolism. On arrival, the patient was hemodynamically unstable with a heart rate of 125 beats/min and hypotensive with a blood pressure of 88/54 mmHg. On physical exam, he was found to be tachypneic, noted to have conjunctival pallor, and was tachycardic with no murmurs, gallops, or rubs appreciated. 

**Figure 1:**
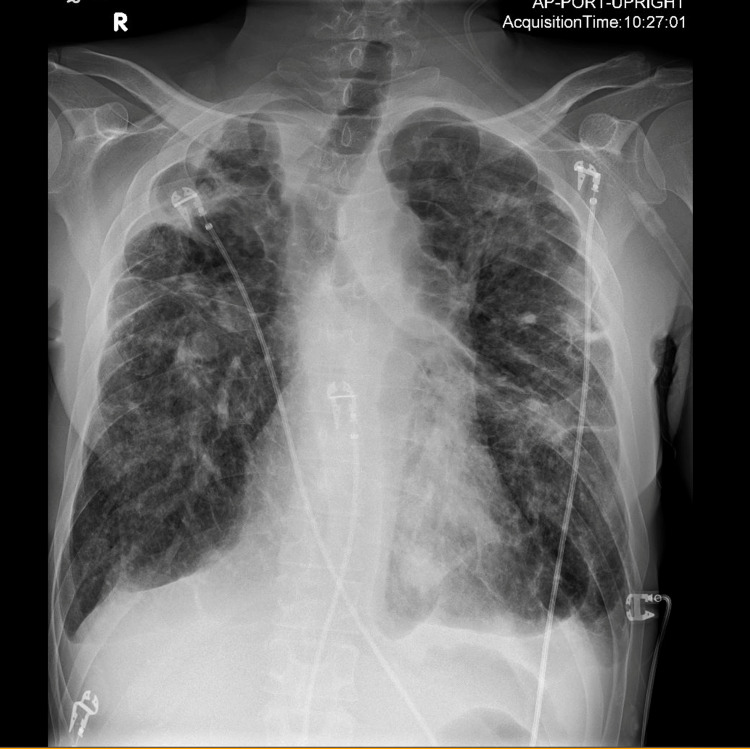
Chest X-ray, anterior-posterior view, demonstrating diffuse emphysematous and fibrotic changes consistent with advanced sarcoidosis

On initial laboratory evaluation, an arterial blood gas showed a pH of 7.17, pCO2 of 94 mmHg, pO2 of 95 mmHg, and HCO3 of 34 mmol/L. He was found to be anemic with a hemoglobin of 7.3 g/dL compared to a prior baseline hemoglobin of 12 g/dL approximately one month prior. Further chemistries revealed a blood urea nitrogen (BUN) of 42 mg/dL and a creatinine of 1.3 mg/dL. A chest X-ray demonstrated diffuse emphysematous and fibrotic changes consistent with advanced sarcoidosis (Figure 1).

Given his respiratory distress, the patient was started on bilevel positive airway pressure (BiPAP). He was transfused with packed red blood cells, improving his hypotension and tachycardia. With suspicion of an upper gastrointestinal bleed in the setting of recent apixaban initiation, intravenous pantoprazole twice daily was started. Following initial stabilization, an esophagogastroduodenoscopy (EGD) identified a 9 mm duodenal ulcer as the source of hemorrhage, necessitating cessation of anticoagulation. 

**Figure 2:**
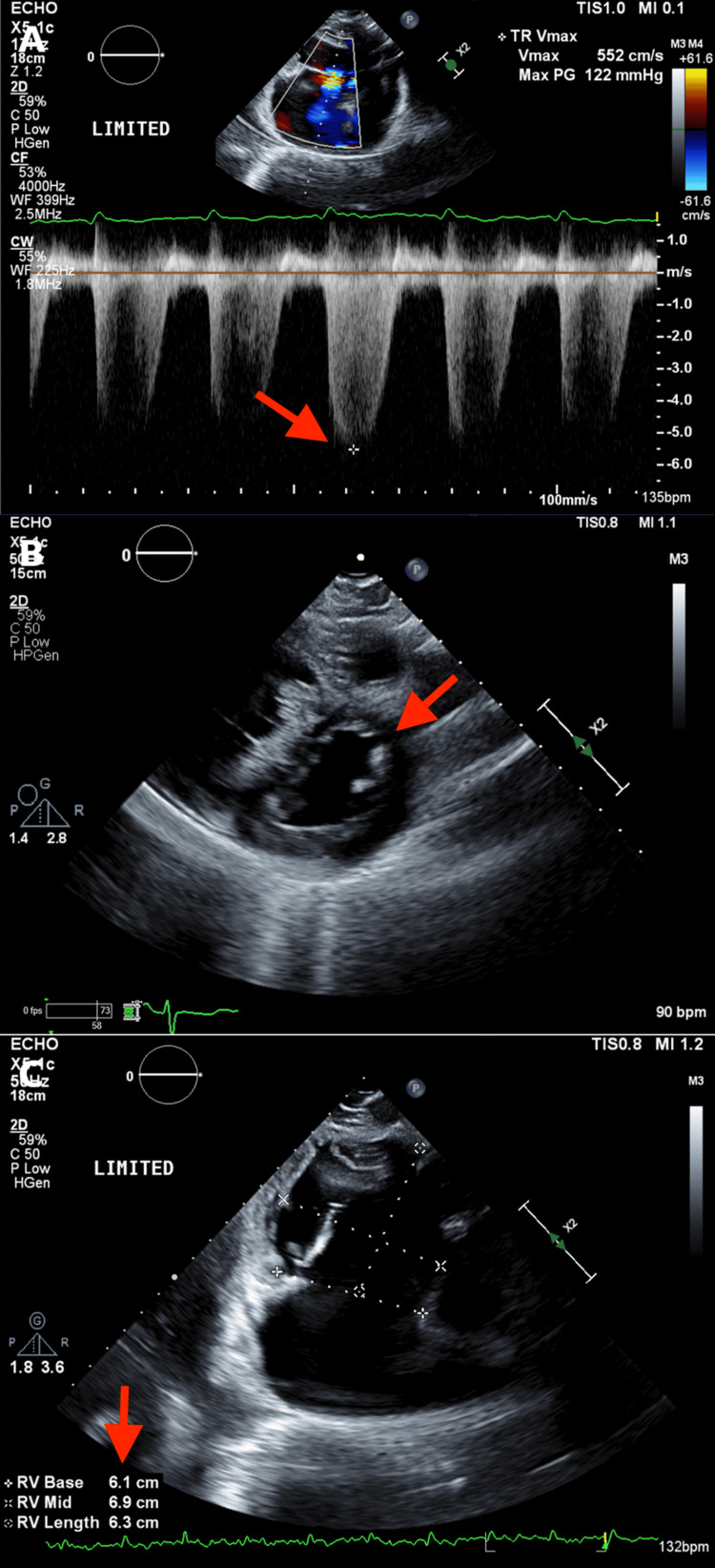
Transthoracic echocardiogram (A) Continuous-wave Doppler across the tricuspid valve showing markedly elevated tricuspid regurgitant velocity (Vmax 5.52 m/s), corresponding to a severely elevated estimated right ventricular systolic pressure. (B) Parasternal short-axis view at the level of the left ventricle demonstrating interventricular septal flattening during systole, consistent with RV pressure overload (D-shaped left ventricle). (C) Apical four-chamber view demonstrating severe right ventricular dilatation with increased RV basal, mid, and longitudinal dimensions. RV: right ventricle; LV: left ventricle; TR: tricuspid regurgitation; Vmax: maximum velocity; PG: pressure gradient

As the gastrointestinal bleed was managed with no improvement in respiratory status, the focus shifted. A limited transthoracic echocardiogram (TTE) demonstrated a reduced left ventricular ejection fraction of 35%. It also showed a severely dilated right ventricle with a severely reduced systolic function. With moderate tricuspid regurgitation, the estimated pulmonary arterial systolic pressure was calculated to be greater than 130 mmHg (Figure 2).

**Figure 3:**
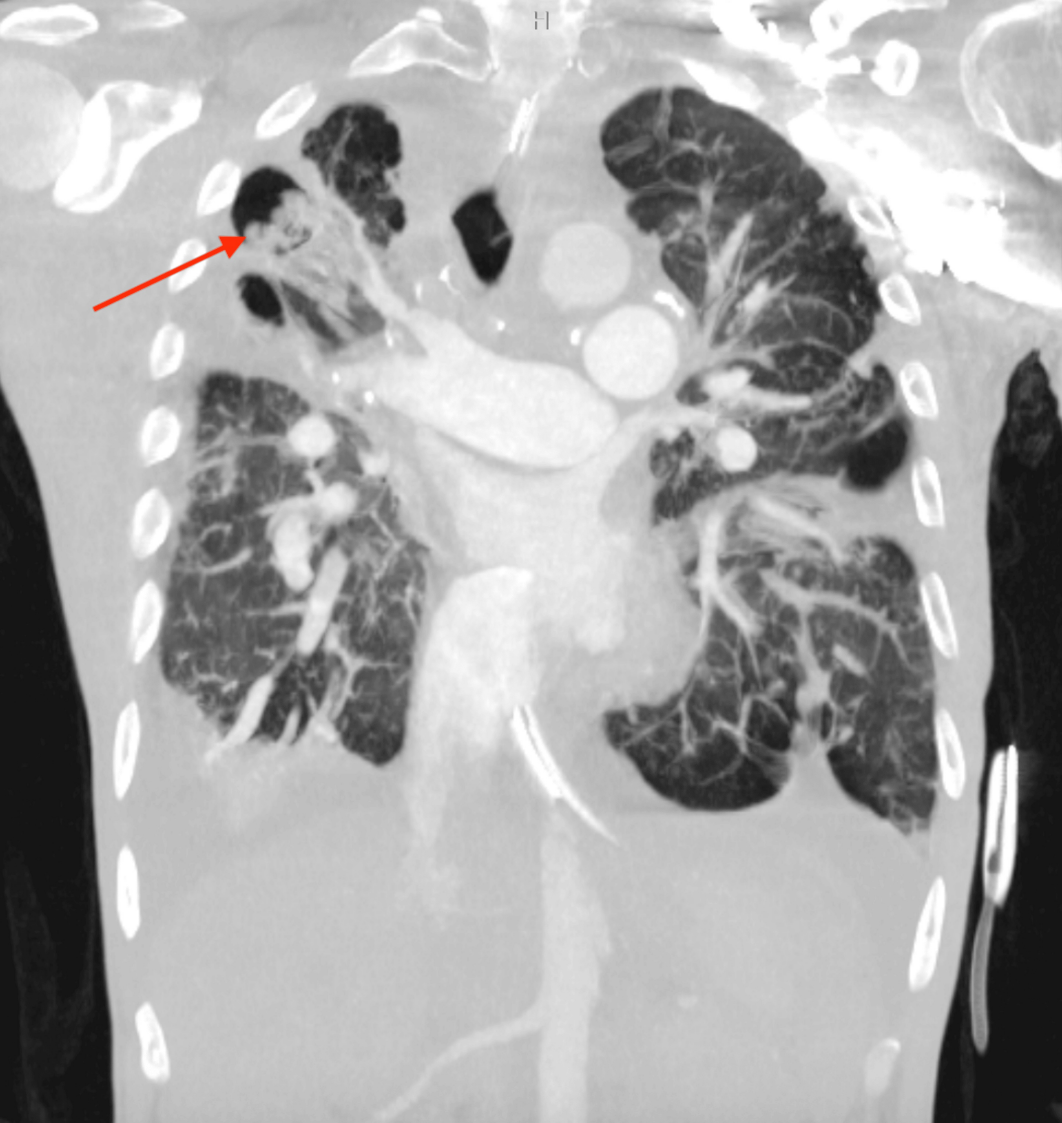
Computed tomography of the chest, anterior-posterior view, demonstrating fibrotic changes and a right upper lobe cavitation

Subsequent imaging with computed tomography (CT) further delineated a complex, multifactorial etiology. CT of the chest confirmed fibrotic changes and a right upper lobe cavitation. Notably, CT imaging also revealed small-caliber pulmonary veins and a suspected chronic occlusion of a right-sided pulmonary artery. Moderate bilateral pleural effusions with associated passive atelectasis were also noted (Figure 3).

The diagnosis was solidified by right heart catheterization, which revealed a mean pulmonary arterial pressure of 71 mmHg (pulmonary arterial systolic pressure 109 mmHg/pulmonary arterial diastolic pressure 54 mmHg) and an elevated mean right atrial pressure of 15 mmHg. The hemodynamic profile confirmed mixed PH: a pulmonary arterial wedge mean pressure of 17 mmHg (Group 2 contribution), a markedly widened transpulmonary gradient of 54 mmHg, and a diastolic pressure gradient of 37 mmHg. 

**Figure 4:**
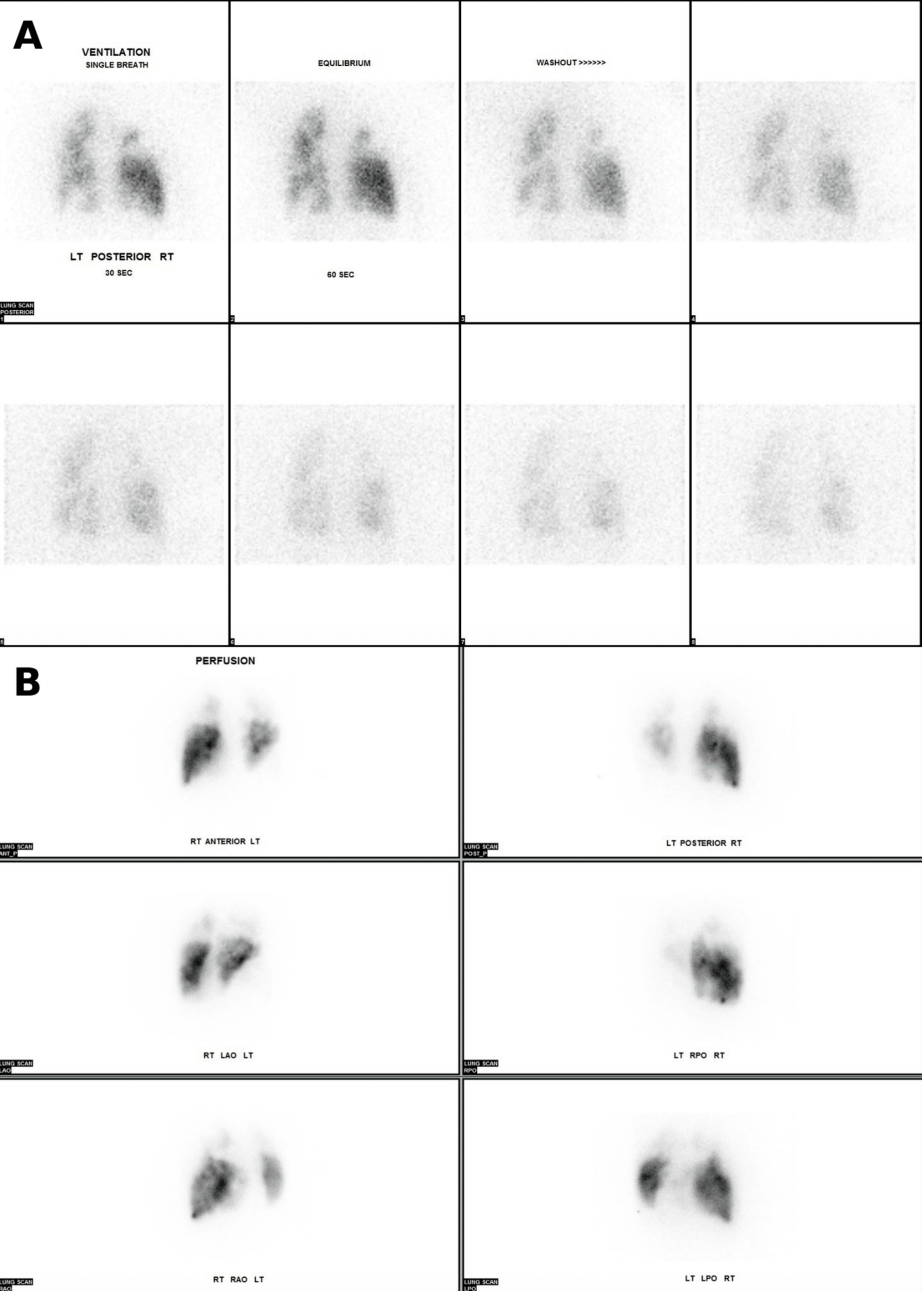
Ventilation-perfusion scan demonstrating mismatched segmental perfusion defects (A) Ventilation images obtained following the inhalation of radiotracer demonstrating relatively preserved and symmetric tracer distribution throughout both lungs without significant segmental defects. (B) Perfusion images revealing multiple segmental perfusion defects, most prominently involving the right lung, with areas of reduced radiotracer uptake that are not matched by ventilation abnormalities. LT: left; RT: right; LAO: left anterior oblique; RAO: right anterior oblique; RPO: right posterior oblique; LPO: left posterior oblique

A subsequent ventilation-perfusion (V/Q) scan was obtained, given evidence of a chronic pulmonary embolism. This was performed as there was a high suspicion of CTEPH. It showed multiple segmental defects with mismatched ventilation and perfusion (Figure 4).

Following the stabilization of the gastrointestinal hemorrhage and confirmation of hemostasis, anticoagulation with apixaban was cautiously resumed. Given the severity of the patient's multifactorial PH and deteriorating clinical status, he was subsequently transferred to a quaternary care center for lung transplant evaluation.

## Discussion

This case illustrates a rare but clinically critical example of severe multifactorial PH, in which overlapping WHO Group 2, Group 4, and Group 5 mechanisms culminate in severe pulmonary pressures and advanced right ventricular failure. While PH classification provides a structured diagnostic framework, SAPH often defies discrete categorization due to its overlapping pathophysiology.

SAPH is one of the most complex etiologies of pulmonary vascular disease. It has been reported in approximately 3-20% of sarcoidosis patients, with prevalence increasing significantly in advanced fibrotic disease [5,6]. In these patients, several mechanisms often overlap, including pulmonary fibrosis with hypoxic vasoconstriction, extrinsic vascular compression, and pulmonary veno-occlusive disease [6]. This patient demonstrated several of these features, including advanced parenchymal disease and heart failure, consistent with a significant Group 5 component.

Importantly, sarcoidosis is increasingly recognized as a prothrombotic condition that predisposes patients to venous thromboembolism and, in some cases, CTEPH [7]. The multiple mismatched perfusion defects on V/Q scanning in this patient are consistent with Group 4 PH, adding another major driver of elevated pulmonary vascular resistance. CTEPH, a potentially treatable cause of PH, requires prompt recognition and specialized evaluation for pulmonary endarterectomy, balloon pulmonary angioplasty, or medical therapy [8].

In parallel, this patient also had HFrEF, contributing to a post-capillary component. Group 2 PH is the most common form of PH globally and is driven by elevated left-sided filling pressures, leading to pulmonary venous congestion and subsequent vascular remodeling [8]. The elevated pulmonary capillary wedge pressure in this case confirms this contribution. Notably, the markedly elevated transpulmonary (54 mmHg) and diastolic pressure gradients (37 mmHg) indicate a combined pre- and post-capillary PH phenotype, which is associated with worse outcomes compared to isolated post-capillary disease [9].

Thus, the coexistence of these mechanisms creates pulmonary vascular injury, characterized by mechanical obstruction from CTEPH, inflammatory and fibrotic remodeling from sarcoidosis, and venous congestion with secondary arteriopathy from HFrEF. Such overlap syndromes are increasingly recognized but remain underrepresented in clinical trials, leading to therapeutic uncertainty. Current literature emphasizes that SAPH management must be individualized and mechanism-driven, often necessitating multidisciplinary collaboration [5]. Importantly, immunosuppressive therapy alone is frequently insufficient to reverse PH in sarcoidosis, especially after vascular remodeling is established [10].

This case also underscores the importance of right heart catheterization in delineating the relative contributions of pre- and post-capillary disease. Noninvasive imaging alone would not have captured the severity of the hemodynamic derangements or the extent of the vascular remodeling.

Finally, the patient's clinical course highlights a critical therapeutic challenge. There are competing risks of anticoagulation for CTEPH and active gastrointestinal bleeding. This dilemma often complicates management in real-world settings and may delay definitive therapy for thromboembolic disease, ultimately contributing to worse outcomes.

## Conclusions

This case highlights a rare presentation of multifactorial PH resulting from the convergence of sarcoidosis (Group 5), chronic thromboembolic disease (Group 4), and HFrEF (Group 2). These processes led to a combined pre- and post-capillary phenotype characterized by extreme pulmonary vascular remodeling, suprasystemic pressures, and advanced right ventricular failure. It underscores the limitations of the traditional WHO classification in complex, overlapping syndromes driven by multiple pathophysiologic mechanisms. Accurate diagnosis requires a high index of suspicion and comprehensive hemodynamic assessment, with right heart catheterization remaining essential for defining disease physiology. Management must be individualized and mechanism-directed, often requiring multidisciplinary collaboration. This case also emphasizes the clinical challenge of balancing anticoagulation for thromboembolic disease in the setting of active bleeding, which may delay therapy and worsen outcomes. Early recognition and referral to specialized centers are critical, although prognosis remains poor once advanced vascular remodeling and right ventricular failure develop.
